# Does the Thalamo-Cortical Synchrony Play a Role in Seizure Termination?

**DOI:** 10.3389/fneur.2015.00192

**Published:** 2015-09-01

**Authors:** Elisa Evangelista, Christian Bénar, Francesca Bonini, Romain Carron, Bruno Colombet, Jean Régis, Fabrice Bartolomei

**Affiliations:** ^1^Service de Neurophysiologie Clinique, CHU Timone, Assistance Publique des Hôpitaux de Marseille, Marseille, France; ^2^UMR1106, INSERM, Marseille, France; ^3^Institut de Neurosciences des Systèmes Marseille, Aix Marseille Université, Marseille, France; ^4^Service de Neurochirurgie Fonctionnelle et Stéréotaxie, Assistance Publique des Hôpitaux de Marseille, Marseille, France

**Keywords:** focal seizures, thalamus, intracerebral EEG, synchrony, seizure termination

## Abstract

The mechanisms underlying seizure termination are still unclear despite their therapeutic importance. We studied thalamo-cortical connectivity and synchrony in human mesial temporal lobe seizures in order to analyze their role in seizure termination. Twenty-two seizures from 10 patients with drug-resistant mesial temporal lobe epilepsy undergoing pre-surgical evaluation were analyzed using intracerebral recordings [stereoelectroencephalography (SEEG)]. We performed a measure of SEEG signal interdependencies (non-linear correlation), to estimate the functional connectivity between thalamus and cortical regions. Then, we derived synchronization indices, namely global, thalamic, mesio-temporal, and thalamo-mesio temporal index at the onset and the end of seizures. In addition, an estimation of thalamic “outputs and inputs” connectivity was proposed. Thalamus was consistently involved in the last phase of all analyzed seizures and thalamic synchronization index was significantly more elevated at the end of seizure than at the onset. The global synchronization index at the end of seizure negatively correlated with seizure duration (*p* = 0.045) and in the same way the thalamic synchronization index showed an inverse tendency with seizure duration. Six seizures out of twenty-two displayed a particular thalamo-cortical spike-and-wave pattern at the end. They were associated to higher values of all synchronization indices and outputs from thalamus (*p* = 0.0079). SWP seizures displayed a higher and sustained increase of cortical and thalamo-cortical synchronization with a stronger participation of thalamic outputs. We suggest that thalamo-cortical oscillations might contribute to seizure termination via modulation of cortical synchronization. In the subgroup of SWP seizures, thalamus may exert a control on temporal lobe structures by inducing a stable hypersynchronization that ultimately leads to seizure termination.

## Introduction

Epileptic seizures are paroxysmal recurrent and self-limiting events, lasting no more than a few minutes. Significant research efforts have directed toward a better understanding of the electrophysiological and neurodynamic correlates of seizure initiation, propagation, and termination (1–6). Nevertheless, at present, the intrinsic mechanisms underlying seizure termination are still unclear despite their therapeutic importance. Identifying mechanisms that result in seizure termination would lead to the development of novel and more efficient interventional approaches aimed to induce or reproduce the same termination mechanisms that are triggered in self-limited seizures.

It is known that mechanisms of seizure termination range in scale from the regulation of transmembrane potentials at the level of single neurons to local neuron and interneuron networks and to long-range cortico-subcortical modulating networks comprising remote structures, such as the brain stem and basal ganglia. These long-range interactions may be reflected by an increased synchronization between distant cortical regions and between cortex and subcortical regulatory circuits during seizures (7).

The exact relationship between synchronization and seizure termination is not completely understood. In an extensive series of *in vivo* cat experiments including multi-site intracellular and extracellular as well as local cortical field potential recordings, it has been observed that there was a greater increase in intrahemispheric synchrony immediately before seizure termination (5, 8). Similarly, in recent studies on human partial seizures utilizing different mathematical methods, cortical correlation increased before the seizure ended (9, 10). It has been thus suggested that the hypersynchrony observed at the end of seizures may play a crucial role in seizure termination.

The role of subcortical structures in modulating seizure termination remains an unanswered question. Subcortical structures play a significant role in modulating seizure activity, in terms of cortical seizure threshold, duration, or severity. Yet, the role of synchronized thalamo-cortical oscillations has been well demonstrated in animal models of absence seizures, where thalamus was shown to provide a resonant circuitry that organizes, amplifies, and synchronizes seizure activity (4, 11).

The involvement of thalamus during partial seizures has been previously described in animal models of temporal lobe seizures (12–18). Electrophysiological recordings of spontaneous limbic seizures in chronic epileptic rats showed that circuits involving the midline thalamus and limbic structures are activated in the early stages of seizure and that the infusion of lidocaine in thalamic nuclei shortens seizure duration (16). Thus, authors suggested that thalamus may be necessary to amplify and distribute ictal activity throughout the system and may play a significant role in seizure modulation.

Regarding the role of thalamus in human partial epilepsies, morphologic and functional neuroimaging studies have found structural alterations (19–23) and metabolic or perfusion changes (24–28) of the ipsilateral thalamus predominant in the dorsomedian nucleus.

Moreover, intracerebral recordings showed that synchronized loops between thalamus and temporal structures take place during the course of seizure and may play a role in amplifying the spreading of the discharge (29).

Based on the notion that thalamus synchronizes its activity with the cortex and on the findings of an increase of synchronization at the end of seizure, one may question the role of the thalamus in seizure termination.

Thus, the purpose of this work was to study the role of the thalamus in seizure termination through its involvement in thalamo-cortical synchronization.

To this aim, we studied thalamic activity in human mesio-temporal lobe seizures (MTLEs) using intracerebral recordings performed with stereoelectroencephalography (SEEG). We analyzed the influence of thalamic inputs and outputs in the last phase of seizures in order to investigate the potential role of thalamus in modulating seizure termination.

## Materials and Methods

### Patient selection and SEEG recordings

**Figure 1:**
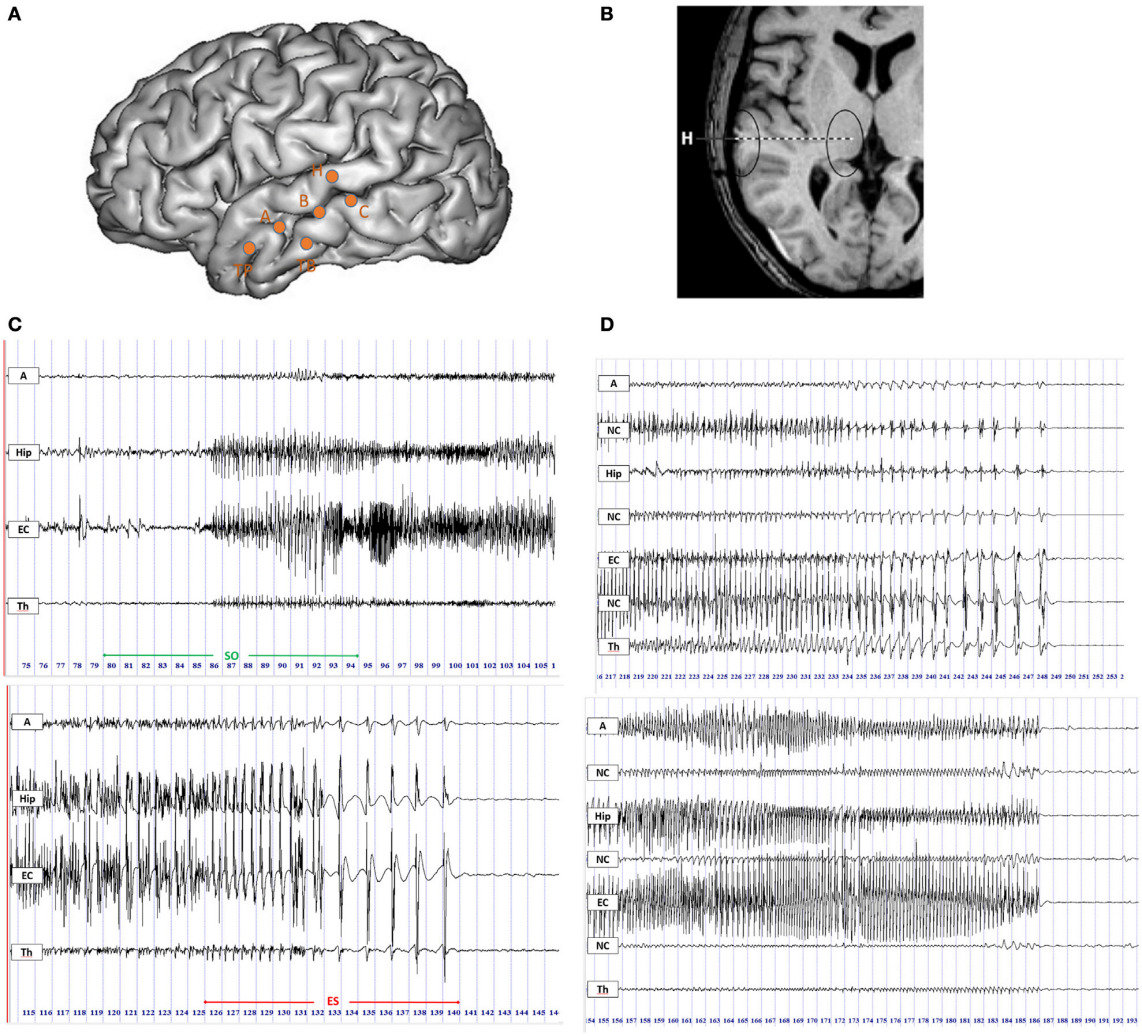
**Intracerebral recordings using multiple contacts electrodes placed according to Talairach’s stereotactic method**. **(A)** Schematic diagram of SEEG electrodes placement on a lateral view of a three-dimensional (3D) reconstruction of the neocortical surface in a patient with MTLE. Electrode A explored the amygdala (internal leads) and the anterior part of the MTG (lateral leads); electrode B explored the anterior hippocampus (internal leads) and the middle part of MTG (lateral leads); electrode TB explored the entorhinal cortex (internal leads) and the anterior part of ITG (external leads); electrode C explored the posterior hippocampus (internal leads) and the posterior part of MTG (lateral leads); electrode H explored the thalamus (internal leads) and the posterior part of STG (lateral leads). **(B)** MRI axial view of standard orthogonal electrode H exploring thalamus by its internal leads and STG by its external leads. **(C)** Ictal SEEG recordings with selected traces from mesio-temporal structures, i.e., amygdala (A), hippocampus (Hip), entorhinal cortex (EC), and from thalamus (Th). Seizure onset period (SO) included 5 s before and 10 s after the onset of high-frequency activity in mesial temporal structures (top panel); the end of seizure period (ES) was defined as the last 15 s of the seizure discharge (bottom panel). **(D)** SEEG recordings of two seizures in the ES period displaying the thalamic rhythmic pattern with a spike-wave activity (on the top) or an arched morphology (on the bottom) coincidental with cortical spike-and-wave discharges. A, amygdala; Hip, hippocampus; EC, entorhinal cortex; NC, neocortex; Th, thalamus.

Twenty-two MTLEs from 10 patients undergoing stereotactic EEG recordings (SEEG) were studied. All patients had a pre-surgical evaluation including detailed clinical evaluation, routine MRI, and surface video-EEG (30). Patients were retrospectively selected for the present study according to the following criteria: (i) the epileptogenic zone is located in the mesial temporal lobe; (ii) at least one intracerebral electrode reached the thalamus. During all SEEG procedures, patients signed informed consent, and the study was approved by the Institutional Review board (IRB00003888) of INSERM (IORG0003254, FWA00005831). Intracranial EEG electrodes were implanted stereotaxically according to the Talairach’s method (31). The location of each electrode contact was based on CT-scan/MRI data fusion according to previous studies (32). The location of the contacts in the thalamus was based on Talairach (33) and Schaltenbrand (34) atlases. Thalamic target was mainly the medial pulvinar group or the posterior part of the dorsomedian nucleus and corresponded to the most internal contact of the electrode passing through the superior temporal gyrus (electrode H, Figure 1) (29, 35). This type of electrode was implanted for clinical purpose, mainly for exploring the superior temporal gyrus and the insula. Signals were recorded on a 128 channel Deltamed™ system, sampled at 256 Hz with no digital filter. Two hardware filters are a high-pass filter (cut-off frequency equal to 0.16 Hz at −3 dB) and a first-order low-pass filter (cut-off frequency equal to 97 Hz at −3 dB) to avoid aliasing.

Patients’ clinical data are provided in Table 1.

**Table 1:** **Clinical data of patients included in the study**.

Patients	1	2	3	4	5	6	7	8	9	10
Age (years)	33	32	22	14	36	35	17	50	54	36
Gender	F	M	M	F	F	F	M	F	M	M
Age at onset (years)	3	17	12	3	16	3	5	15	35	18
Epilepsy duration (years)	30	15	10	11	20	32	12	35	19	18
Etiology	Unk	CD	As	HS	HS	CD	HS	Non-specific	Unk	Unk
Aura	Deja vu	Anxiety, olfactive	Deja vu	No	No	Epigastric, cold	No	Cold, fear, taste	No	Anxiety
Early loss of contact	Yes	Yes	Yes	Yes	No	No	Yes	Yes	Yes	Yes
Secondary generalization	Rare	Frequent	Rare	No	Rare	Rare	Rare	No	Frequent	Rare
Type of TLE	L-MTLE	L-MTLE	R-MLTLE	L-MTLE	R-MTLE	R-MTLE	L-MTLE	R-MTLE	L-MTLE	L-MLTLE

*M, male; F, female; As, astrocytoma affecting the lateral temporal cortex; CD, mesial temporal cortical dysplasia; HS, hippocampal sclerosis; MTL, mesial temporal lobe; Unk, unknown; TLE, temporal lobe epilepsy; L-MTLE, left mesial temporal lobe epilepsy; R-MTLE, right mesial temporal lobe epilepsy*.

### Procedure: SEEG signal analysis

#### Definition of Regions

We analyzed the statistical interdependencies between bipolar signals exploring different regions of interest. We selected the contacts exploring in each patient three mesial temporal regions (amygdala, hippocampus, and entorhinal cortex, respectively A, Hip, and EC), three neocortical temporal regions (NC, corresponding to the superior, middle, or inferior temporal gyrus, respectively STG, MTG, and ITG) and the thalamus (Th) (Figures 1A,B). Electrodes with significant artifacts were excluded.

### Periods of interest

For each seizure, two periods of interest (both lasting 15 s) were selected (Figure 1C):

- *Seizure onset (SO)* including 5 s before and 10 s after the appearance of a tonic discharge in mesial temporal structures.
- *End of seizure (ES)* including the last 15 s of the seizure discharge.

Duration of each seizure was calculated in seconds starting from the beginning of SO to the end of ES.

#### “Thalamic” Patterns

Firstly, intracerebral signals were visually inspected. In the ES period, 6 out of 22 seizures displayed an electrical pattern characterized by rhythmic activity with a spike-wave or an arched morphology coincidental with a cortical spike-and-wave discharges (SWDs) (Figure 1D). This pattern was also frequently characterized by a gradual increase in the inter-spike duration (4/6 seizures). A similar thalamic pattern has been previously described by Meeren and colleagues (4) during the generalized SWDs in a genetic model of absence epilepsy, the WAG/Rij rat. We defined this spike-and-wave pattern (SWP) as pattern “A”. The other 16 seizures with different electrical patterns from pattern A were named pattern “B”.

### Estimation of signal correlation

Non-linear regression analysis (estimation of *h*^2^ coefficient) (36) was used to study functional connectivity between thalamus and selected cortical regions (A, Hip, EC, and NC). This method gives an estimation of the degree and the direction of the link between brain regions. Main practical applications of this method have concerned epileptic activity analysis both in animals and in humans (2, 4, 37, 38). This method is a pair-wise approach, where the amplitude of a signal *Y* is described as a function of the amplitude of a signal *X* using a non-linear regression curve and to compute the variance of *Y* that is explained, or predicted, by *X* according to this regression curve. A numerical approximation of the non-linear regression curve is obtained by describing the scatterplot of *Y* vs. *X* by segments of linear regression curves (i.e., this is a piecewise linear model).

The non-linear correlation coefficient *h*^2^ is computed and takes its values in [0,1]. Low values of *h*^2^ denote that signals *X* and *Y* are independent. On the opposite, high values of *h*^2^ mean that signal *Y* may be explained by a continuous transformation (possibly non-linear) of signal *X*, i.e., signals *X* and *Y* are dependent.

Time delay between signals *X* and *Y* was also calculated. Signals were sampled at 256 Hz and a 2 s window sliding with steps of 1 s were chosen. This resulted in a connectivity graph, with each node corresponding to a region of interest. Between two nodes, we selected the higher *h*^2^ value between *X* → *Y* and *Y* → *X*, and the sign of the corresponding time delay defined the direction of the connection.

**Figure 2:**
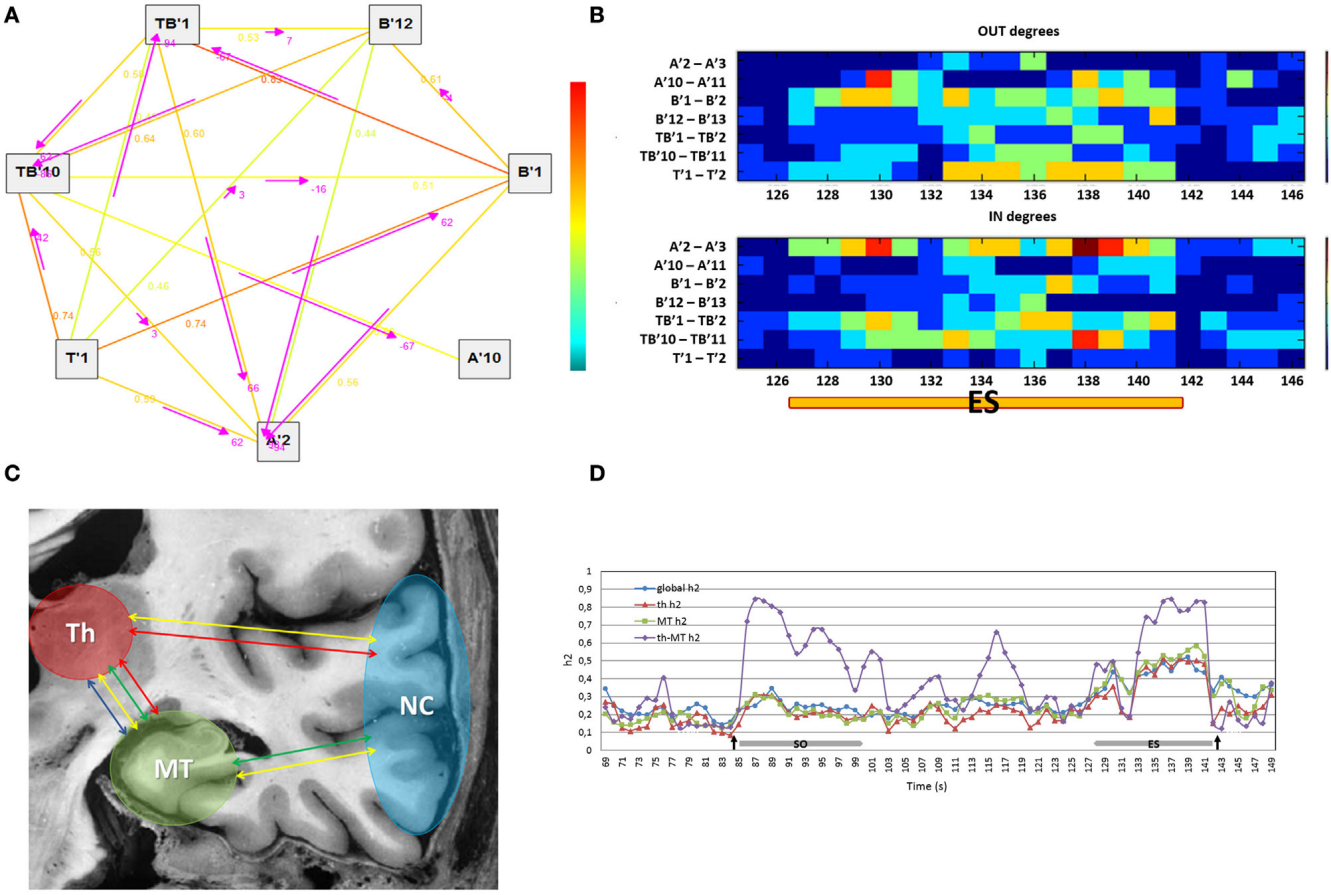
**(A)** The graph shows the direction, represented by the arrows, of functional connections between the regions of interest selected (on the left) in a 2 s window of an ictal SEEG recording (on the right). The strength of connections is indicated by the numerical values (*h*^2^ values) scaling from 0 to 1 and by a colored scale (red representing the maximum degree and blue the minimum). **(B)** The colored graph displayed the variations of output and input connection measures for each region compared to the others during the end of seizure period (ES) in one MTLE seizure. A′2–A′3: internal contacts of the electrode exploring the left amygdala; A′10–A′11: external contacts of the same electrode exploring the anterior part of the left MTG; B′1–B′2: internal contacts of the electrode exploring the left anterior hippocampus; B′12–B′13: external contacts of the same electrode exploring the middle part of the left MTG; TB′1–TB′2: internal contacts of the electrode exploring the left entorhinal cortex; TB′11–TB′12: external contacts of the same electrode exploring the anterior part of the left ITG; T′1–T′2: internal contacts of the electrode exploring left thalamus. **(C)** Connectivity measures on regions of interest: global connectivity measure (yellow arrow), thalamic connectivity measure (red arrow), mesio-temporal connectivity measure (green arrow) and thalamo-mesio temporal connectivity measure (blue arrow). Th, thalamus; MT, mesio-temporal regions; NC, neocortical regions. **(D)** Variations in connectivity measure profiles during the course of one patient’s seizure. Arrows pointed respectively to the onset and the end of seizure. Global *h*^2^, global connectivity measure; th *h*^2^, thalamic connectivity measure; MT *h*^2^, mesio-temporal connectivity measure; th-MT *h*^2^, thalamo-mesio temporal connectivity measure; ES, end of seizure; SO, seizure onset.

In this work, we propose to use a network measure that estimates the number of output and input connections for each node of the graph (in graph theory terminology, these are the output and input degrees, respectively). Output and input degrees were computed for each region compared to the others combining the number of functional connections with *h^2^* values higher than 0.4 (in order to take into account only meaningful connections) and the time delay between signals. A visual representation of output and input connectivity was displayed in colored graphs showing the variations of directionality measures during the course of seizures (Figures 2A,B). To the aim of this work, we specifically focused on the thalamic and mesio-temporal outputs and inputs; thalamic output and input measures refer to functional connections between the thalamus and other cortical regions explored; similarly, mesio-temporal output and input measures refer to functional connections between a mesio-temporal region and other regions explored, included thalamus. Computations were done with the Anywave freeware (39) available at http://meg.univ-amu.fr.

### Statistical analysis

#### Synchronization Indices

In order to obtain a global connectivity measure, an average of *h*^2^ values of all pairs of signals was computed for each analysis window (2 s sliding window with steps of 1 s) on regions of interest. Thus, we computed a thalamic connectivity measure by averaging the *h*^2^ values obtained from the interactions between the thalamus and cortical regions. A mesio-temporal connectivity measure was obtained by averaging the *h*^2^ values between mesio-temporal structures and the remaining regions (including thalamus). Finally, a thalamo-mesio temporal connectivity measure was derived by averaging the *h*^2^ values obtained from the interaction between thalamus and mesio-temporal regions (Figure 2C). All connectivity measures were estimated as a function of time during seizures as shown in Figure 2D.

We then performed a second average across time either in the seizure onset period (SO) and in the end of seizure period (ES). For each period, we computed the average across a 3 s window around the peak of connectivity, resulting in a global index, a thalamic index (Th index), a mesio-temporal index (MT index), and a thalamo-mesio temporal index (Th-MT index).

#### Graph Measures

Similarly to the synchronization indices, we computed the average of thalamo and mesio-temporal output and input measures in the ES period. This resulted in graph measures in the ES period, specifically thalamic output and input measures (Th OUT, Th IN) and mesio-temporal output and input measures (MT OUT, MT IN).

#### Statistical Tests

In order to analyze how thalamus, mesio-temporal regions, and neocortex synchronize their activity during the course of seizures, global, thalamo, mesio-temporal, and thalamo-mesio temporal synchronization indices (global index, Th index, MT index, and Th-MT index) were compared between the SO and ES period by a Wilcoxon test.

A non-parametric correlation test (Spearman test) was performed to find possible correlations between synchronization indices in ES period (global index, Th index, MT index, and Th-MT index) and seizure duration.

In order to compare seizure duration, synchronization, and direction indices between the two groups (pattern A and B), a non-parametric Mann–Whitney test has been applied.

Electrophysiological data are provided in Table 2.

**Table 2:** **Electrophysiological data**.

Patients	Seizures	Pattern	Seizure duration(s)	ES global index	ES Th index	ES MT index	ES Th-MT index	SO global index	SO Th index	SO MT index	SO Th-MT index	Th OUT	Th IN	MT OUT	MT IN
1	1	A	55	0.49	0.51	0.52	0.82	0.19	0.22	0.24	0.51	4.25	0.38	3.13	1.5
	2	A	90	0.48	0.5	0.53	0.81	0.22	0.25	0.18	0.20	2.17	1.00	3.67	1.00
2	3	A	150	0.46	0.52	0.42	0.47	0.17	0.12	0.17	0.14	3.30	2.20	1.00	3.50
	4	B	540	0.26	0.21	0.24	0.2	0.15	0.10	0.22	0.14	1.18	0.73	1.55	0.18
3	5	B	59	0.38	0.41	0.34	0.32	0.26	0.22	0.30	0.21	0.50	0.40	1.60	0.60
	6	B	63	0.33	0.36	0.33	0.42	0.19	0.16	0.24	0.13	0.56	1.22	0.56	0.44
	7	B	88	0.44	0.45	0.44	0.36	0.22	0.17	0.26	0.18	1.90	1.80	3.00	1.70
4	8	A	60	0.59	0.55	0.59	0.6	0.19	0.12	0.15	0.10	2.91	1.64	2.55	1.82
	9	A	53	0.49	0.53	0.5	0.53	0.22	0.14	0.19	0.13	0.67	3.17	2.67	0.08
5	10	A	70	0.49	0.58	0.42	0.47	0.14	0.12	0.10	0.13	2.00	1.86	1.29	2.00
	11	B	126	0.41	0.43	0.4	0.52	0.13	0.14	0.11	0.12	1.75	1.75	2.63	1.25
6	12	B	54	0.36	0.33	0.32	0.29	0.15	0.10	0.12	0.09	0.8	0.70	2.10	0.80
	13	B	65	0.35	0.37	0.43	0.45	0.17	0.11	0.17	0.12	0.22	1.78	3.33	0.22
7	14	B	200	0.33	0.33	0.38	0.57	0.13	0.12	0.13	0.11	1.50	1.70	2.40	1.00
	15	B	290	0.29	0.29	0.32	0.4	0.17	0.15	0.16	0.18	0.5	0.50	0.17	1.33
8	16	B	455	0.27	0.27	0.34	0.25	0.11	0.12	0.13	0.29	2.20	0.20	2.20	0.60
	17	B	70	0.27	0.34	0.39	0.42	0.14	0.18	0.16	0.47	0.25	1.25	1.63	0.38
9	18	B	113	0.31	0.33	0.34	0.55	0.11	0.14	0.10	0.14	1.11	0.78	1.22	0.78
	19	B	124	0.31	0.42	0.35	0.54	0.16	0.17	0.16	0.19	1.89	0.89	1.22	1.78
10	20	B	64	0.46	0.38	0.42	0.46	0.37	0.28	0.45	0.37	0.60	1.20	1.30	0.80
	21	B	50	0.37	0.43	0.39	0.38	0.32	0.26	0.42	0.35	0.70	2.50	2.30	0.20
	22	B	55	0.28	0.38	0.31	0.37	0.22	0.17	0.20	0.12	1.00	1.60	1.70	0.30

*Global index, global synchronization index; Th index, thalamic synchronization index; MT index, mesio-temporal synchronization index; Th-MT index, thalamo-mesio temporal synchronization index; ES, end of seizure period; SO, seizure onset period; Th OUT, thalamic output measure; Th IN, thalamic input index; MT OUT, mesio-temporal output measure; MT IN, mesio-temporal input index*.

## Results

### Dynamic changes according to synchronization indices

**Figure 3:**
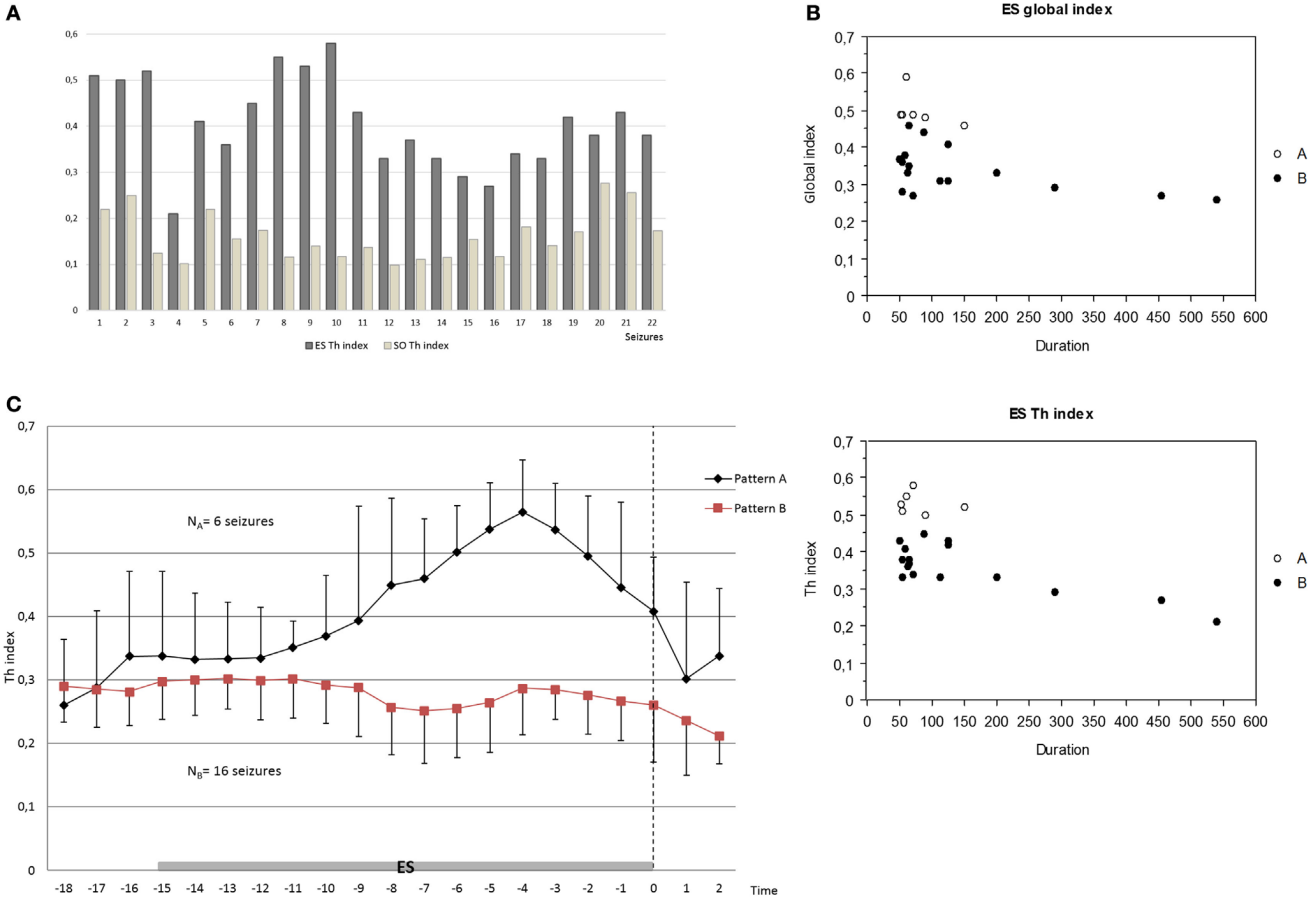
**(A)** Increase of the thalamic synchronization index (Th index) in the ES period compared to the SO period in all seizures analyzed (*p* < 0.0001 for each comparison) by Wilcoxon test. **(B)** Negative correlation between global index and seizure duration (*p* = 0.045) and inverse tendency between Th index and seizure duration (*p* = 0.052, *R*^2^ 0.40) in the ES period by Spearman test. **(C)** Different patterns of the increase of thalamic connectivity between groups A and B in the ES period; pattern A seizures (*N*_A_ = 6) showed an overall stable, higher, and longer increase of thalamo-cortical synchronization clearly before the end of seizure compared to pattern B seizures (*N*_B_ = 16). The mean and the average deviation of values are shown. Dashed arrow indicates the end of seizure.

Significant differences of all synchronization indices were observed between the onset of seizure (SO) and the end of seizure (ES). Indeed, global, thalamo, mesio-temporal, and thalamo-mesio temporal synchronization indices (global index, Th index, MT index, and Th-MT index) displayed a marked increase in the ES period compared to the SO period (*p* < 0.0001 for each comparison, Figure 3A).

In order to estimate the influence of synchronization changes on seizure duration, we tested for a correlation between synchronization indices at ES period (global index, Th index, MT index, and Th-MT index) and duration of seizures.

The global index was negatively correlated with seizure duration (*p* = 0.045), namely higher synchronization values corresponded to lower seizure duration. In the same way, we found a trend for an inverse tendency between Th index and seizure duration (*p* = 0.052, *R*^2^ 0.40) (Figure 3B). In contrast, no significant correlations or tendency were found between other synchronization indices (Th-MT index and MT index) and seizure duration.

### Pattern differences

We then studied how connectivity measures, namely global, thalamic, mesio-temporal, and thalamo-mesio temporal, vary as a function of time during the last phase of seizure. Comparing group A (seizures with thalamic spike-and-wave pattern) with group B (seizures with other thalamic patterns), thalamic connectivity disclosed different courses in the ES period. Indeed, pattern A seizures showed an overall stable, higher, and longer increase of thalamo-cortical synchronization clearly before the end of seizure. On the contrary, pattern B seizures showed a heterogeneous, unstable, and shorter increase of thalamic synchronization, often localized at the beginning of ES period and followed by a slow synchrony decrease (Figure 3C).

**Figure 4:**
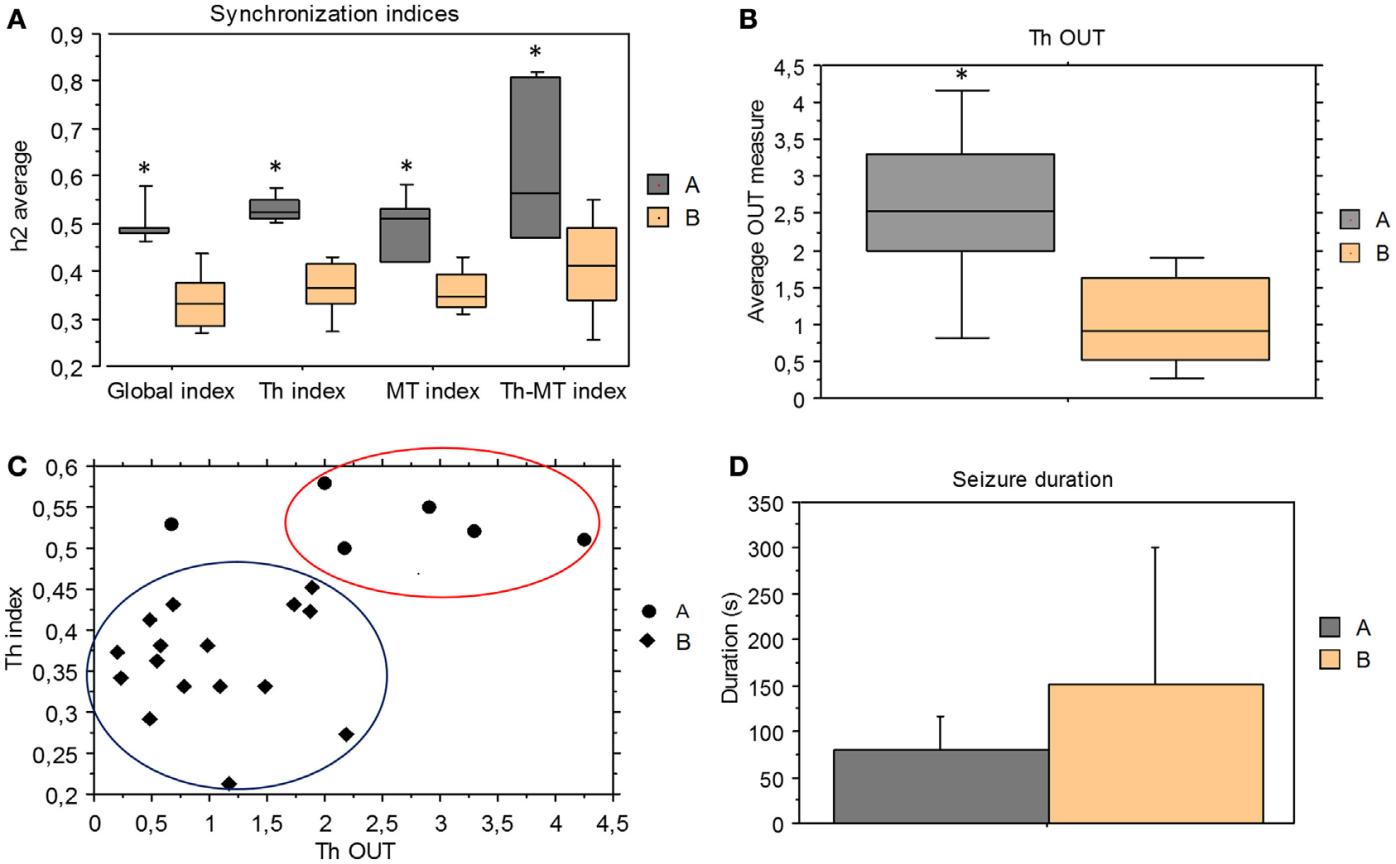
**Comparison between group A and B**. **(A)** Mean and standard deviation of synchronization indices derived from ES period for the two groups; all synchronization indices were significantly higher in pattern A than pattern B seizures (Mann–Whitney test, global index *p* = 0.0004, Th index *p* = 0.0004, Th-MT index *p* = 0.0063, and MT index *p* = 0.0015). **(B)** Mean and standard deviation of thalamic output graph measures (Th OUT) in group A and B (*p* = 0.0079). **(C)** Pattern A seizures showed higher thalamic synchronization indices (Th index) and thalamic output measures (Th OUT) than pattern B seizures in the ES period. **(D)** Mean of seizure duration in group A and group B. No statistical difference between seizure A and seizure B duration, but pattern A group had only short seizures, while pattern B included a wide range of seizure duration.

We compared the synchronization indices and the direction indices derived from ES period between pattern A and pattern B seizures. All synchronization indices were significantly higher in pattern A than pattern B group (global index *p* = 0.0004, Th index *p* = 0.0004, Th-MT index *p* = 0.0063, and MT index *p* = 0.0015) (Figure 4A).

Concerning the direction measures, only thalamic output measure (Th OUT) showed significant higher values (*p* = 0.0079) in pattern A compared with pattern B group (Figure 4B). On the contrary, no significant differences were found comparing thalamic input, mesio-temporal output, and mesio-temporal input measures (Th IN, MT OUT, and MT IN). Therefore, in the last phase of seizure, synchronization levels and thalamic outputs were higher in pattern A than in pattern B (Figure 4C).

Taken as a whole, no statistical significant difference was found in seizure duration between A and B groups but pattern A group included only short seizures, while pattern B comprised a wide range of seizure duration (Figure 4D). Moreover, in two patients showing seizures with both patterns, pattern A seizures were shorter than pattern B seizures (150 s vs. 540 s, 70 s vs. 126 s).

## Discussion

In the present study, we analyzed the ictal functional connectivity between mesio-temporal regions and thalamus by non-linear correlation analysis in 22 drug-resistant MTLEs. The aim of this work was to explore the dynamic involvement of thalamus in partial seizures and its potential contribution in seizure termination.

In all the 22 analyzed mesio-temporal seizures, thalamic activity was affected by the epileptic discharge during the course of seizure. Results of non-linear correlation analysis showed a significant increase of synchronization between thalamus, mesio-temporal region and neocortex (*p* < 0.0001) in the last phase of seizure, compared to the beginning.

These results are in agreement with previous animal and human studies that reported an increase of synchronization at the end of seizure. Indeed, in an extensive series of *in vivo* cat experiments, including multi-site intracellular and extracellular as well as local cortical field potential recordings (5, 8, 40), it has been observed a progressive increase of cortical synchrony during the course of seizure, thus promoting the recruitment of neurons. However, when all the affected neuronal pool was involved into highly synchronous paroxysmal activity, the seizure stopped. As a possible mechanism, the authors proposed that the increased depolarization during the full-blown seizure was associated with the activation of the hyperpolarizing sodium- and calcium-activated potassium currents, overcoming the depolarizing influence of ionic currents. Moreover, an additional contribution may be represented by a dramatic decrease in the extracellular calcium concentration resulting in the decrease of effectiveness of synaptic transmission.

Recent intracranial studies on human partial seizures, making use of different signal interdependencies analysis, described a progressive increase of cortical correlation, maximal at the end of seizure (9, 10, 29, 41).

Although the exact relationship between increase of synchronization and seizure termination is not yet completely understood, the highly synchronous paroxysmal activity in large neuronal networks observed at the end of seizure may be considered as an active seizure termination mechanism.

The role of thalamo-cortical interactions in modulating epileptic activity has been well demonstrated in absence epilepsy. Indeed, highly synchronized thalamo-cortical oscillations represent the neuronal substrate for the generation and maintenance of SWDs and thalamus probably provides a resonant circuitry to amplify and sustain the rhythmic discharges (4).

Although the thalamic involvement in partial seizures has been described in animal models (13–16, 42), its role in modulating the epileptic activity, namely influencing generation, propagation, and termination of seizures, has been less characterized in humans.

Indeed, only few intracerebral studies explored thalamic activity in human partial seizures (29, 35, 43, 44). We previously investigated the thalamic activity during temporal lobe seizures using a non-linear correlation analysis (29). We demonstrated that thalamo-cortical loop takes place during the course of mesiotemporal seizures after a variable latency and tends to be more strongly involved between thalamus and neocortical regions, while seizures started from mesio-temporal regions.

The potential role of thalamus in seizure termination is however not well known. Taken as a whole, subcortical networks, including the thalamo-cortical loop, may modulate different dynamic aspects of epileptic seizure (7, 45).

In this study, we found a constant thalamic recruitment and a marked increase of thalamo-cortical synchronization in the last phase of each analyzed seizure, thus confirming the concept that thalamo-cortical network is highly involved in modulating the epileptic discharge in human mesio-temporal lobe epilepsy.

Moreover, we found an inverse tendency between seizure duration and respectively global and thalamo-cortical synchronization levels at the end of seizure. Therefore, an increase of final global and thalamo-cortical synchronization corresponded to a decrease in seizure duration. We speculated that thalamo-cortical oscillations might exert a seizure terminating role via modulation of cortical synchronization. In fact, thalamo-cortical oscillations promote a progressive increase of hypersynchronous activity in large neuronal networks during the course of seizure, thus likely leading to a massive and simultaneous neuronal hyperpolarization and finally to seizure termination [see Ref. (5)].

Recently, Langlois and colleagues examined the involvement of the thalamic parafascicular nucleus (PF) in the occurrence of hippocampal paroxysmal discharges (HPDs), in *in vivo* intracellular and extracellular as well as local field potential recordings in a chronic mouse model of MTLE. They showed that PF neurons fire synchronously just before the end of the HPDs (18). Moreover, authors demonstrated that the high-frequency PF stimulation (130 Hz) interrupted the ongoing HPS and the pharmacological inhibition or activation of the PF neurons respectively suppresses or aggravates HPDs, suggesting that the PF nucleus plays a role in the active termination of seizure.

The medial pulvinar group (PuM) is the main explored part of the thalamus in our study. Recording this structure does not require electrodes in addition to those exploring regions potentially involved in the epileptogenic zone. Recent intracerebral-evoked responses’ recordings obtained after PuM and cortical electrical stimulation in seven epileptic patients undergoing pre-surgical evaluation, confirmed the developed PuM connectivity with mesial temporal structures (44), as previously reported by anatomical studies in monkeys (46, 47).

Moreover, in human thalamus, PuM is one of the most concentrated nucleus in calbindin matrix cells (48, 49). These thalamo-cortical matrix cells receive inputs from subcortical pathways and project more diffusely upon the cortex. In turn, cortico-thalamic fibers project to new thalamic nuclei and via matrix cells to other cortical areas, supporting the spread of activity (50, 51). Such an organization of connectivity between PuM and cortex would allow the modulation of cortico-cortical networks (52).

By visual inspection of intracerebral ictal activity at the end of seizures, a definite subgroup of seizures displaying a spike-and-wave pattern has been identified (SWP-pattern A seizures). A similar pattern has been previously described in regard to generalized SWDs in animal model of absence epilepsy and reflects the highly synchronized oscillations of thalamo-cortical networks (4). In the last phase of spike-and-wave seizures, we found much higher overall synchronization values, especially for the global and thalamo-cortical synchronization (*p* = 0.0004) compared to seizures with different patterns (pattern B seizures). Moreover, this group displayed a higher and sustained increase of thalamo-cortical synchronization clearly before seizure termination with a stronger participation of thalamic outputs. In addition, we found that this pattern was clearly related to a significant higher value of thalamic output measure (Th OUT), indicating that the driving input in the loop is thalamus.

Although there was not a significant difference in duration between the two groups, the spike-and-wave seizures were always short in duration, while the other pattern seizures varied in a wider range of durations. Furthermore, in patients showing both seizure patterns, spike-and-wave pattern seizures were consistently shorter.

Altogether, these results suggest that MTLEs disclosing this spike-and-wave pattern have stronger thalamo-cortical synchronized activity before the end of seizure, driven by thalamic outputs. Moreover, we speculate that thalamus may in this case exert a control on cortical structures by inducing a stable hypersynchronization that ultimately leads to seizure termination.

Finally, a better understanding of the role of subcortical circuits in partial seizure termination may improve insights into brain autoregulatory mechanisms. Moreover, it may offer new venues for developing novel treatments and for advancing protocols of thalamic deep brain stimulation (DBS) in patients with epilepsy. Recent development of DBS in epilepsy mainly focused on the anterior nucleus (53), showing an efficacy for reducing seizures in a significant percentage of patients. Our data suggest that the PuM could be an interesting target in this perspective.

## Author Contributions

EE and CB contributed to study design, data collection, data analysis, and writing manuscript. FBo and RC contributed to data collection and writing manuscript. RC and JR performed stereotactic explorations of patients. BC has contributed to software development and signal analysis. FBa has contributed to study design, data collection and analysis, and writing manuscript. All authors had reviewed and agreed the final manuscript.

## Conflict of Interest Statement

The authors declare that the research was conducted in the absence of any commercial or financial relationships that could be construed as a potential conflict of interest.
